# Isolated Anastomotic Ulcers Are Associated with a Higher Long-Term Risk for Postoperative Recurrence and a Differential Mucosa-Associated Microbiome Composition in Patients with Crohn’s Disease Following Ileocolic Resection

**DOI:** 10.1093/ibd/izaf147

**Published:** 2025-07-16

**Authors:** Michiel Thomas Jan Bak, Pablo Andres Olivera, Cristian Hernandez-Rocha, Krzysztof Borowski, Williams Turpin, Haim Leibovitzh, Raquel Milgrom, Joanne Stempak, Mark Silverberg, Sun-Ho Lee

**Affiliations:** Zane Cohen Centre for Digestive Diseases, Lunenfeld-Tanenbaum Research Institute, Sinai Health System, 60 Murray St, Toronto, ON M5T 3L9, Canada; Division of Gastroenterology, Mount Sinai Hospital, Sinai Health System, University of Toronto, 600 University Ave, Toronto, ON M5G 1X5, Canada; Department of Gastroenterology and Hepatology, Erasmus University Medical Center Rotterdam, Doctor Molewaterplein 40, 3015GD, Rotterdam, the Netherlands; Zane Cohen Centre for Digestive Diseases, Lunenfeld-Tanenbaum Research Institute, Sinai Health System, 60 Murray St, Toronto, ON M5T 3L9, Canada; Division of Gastroenterology, Mount Sinai Hospital, Sinai Health System, University of Toronto, 600 University Ave, Toronto, ON M5G 1X5, Canada; Zane Cohen Centre for Digestive Diseases, Lunenfeld-Tanenbaum Research Institute, Sinai Health System, 60 Murray St, Toronto, ON M5T 3L9, Canada; Division of Gastroenterology, Mount Sinai Hospital, Sinai Health System, University of Toronto, 600 University Ave, Toronto, ON M5G 1X5, Canada; Zane Cohen Centre for Digestive Diseases, Lunenfeld-Tanenbaum Research Institute, Sinai Health System, 60 Murray St, Toronto, ON M5T 3L9, Canada; Zane Cohen Centre for Digestive Diseases, Lunenfeld-Tanenbaum Research Institute, Sinai Health System, 60 Murray St, Toronto, ON M5T 3L9, Canada; Division of Gastroenterology, Mount Sinai Hospital, Sinai Health System, University of Toronto, 600 University Ave, Toronto, ON M5G 1X5, Canada; Department of Nutritional Sciences Temerty Faculty of Medicine, University of Toronto, 1 King’s College Cir, Toronto, ON M5S 3K3, Canada; Zane Cohen Centre for Digestive Diseases, Lunenfeld-Tanenbaum Research Institute, Sinai Health System, 60 Murray St, Toronto, ON M5T 3L9, Canada; Division of Gastroenterology, Mount Sinai Hospital, Sinai Health System, University of Toronto, 600 University Ave, Toronto, ON M5G 1X5, Canada; Zane Cohen Centre for Digestive Diseases, Lunenfeld-Tanenbaum Research Institute, Sinai Health System, 60 Murray St, Toronto, ON M5T 3L9, Canada; Zane Cohen Centre for Digestive Diseases, Lunenfeld-Tanenbaum Research Institute, Sinai Health System, 60 Murray St, Toronto, ON M5T 3L9, Canada; Zane Cohen Centre for Digestive Diseases, Lunenfeld-Tanenbaum Research Institute, Sinai Health System, 60 Murray St, Toronto, ON M5T 3L9, Canada; Division of Gastroenterology, Mount Sinai Hospital, Sinai Health System, University of Toronto, 600 University Ave, Toronto, ON M5G 1X5, Canada; Zane Cohen Centre for Digestive Diseases, Lunenfeld-Tanenbaum Research Institute, Sinai Health System, 60 Murray St, Toronto, ON M5T 3L9, Canada; Division of Gastroenterology, Mount Sinai Hospital, Sinai Health System, University of Toronto, 600 University Ave, Toronto, ON M5G 1X5, Canada

**Keywords:** Crohn’s disease, anastomotic ulcers, microbiome, postoperative recurrence

## Abstract

**Background:**

The clinical relevance and underlying mechanism of isolated anastomotic ulcers (IAUs) following ileocolic resection (ICR) in patients with Crohn’s disease (CD) are poorly understood. This study aimed to assess the postoperative recurrence (POR) risk and the mucosa-associated microbiome composition in CD patients with or without IAUs among those with a healthy neo-terminal ileum (TI).

**Methods:**

CD patients who underwent ICR and without any ulcerations in the neo-TI (SES-CD ≤2) at first postoperative ileocolonoscopy were identified from an ongoing prospective multicenter study. The primary study outcome was time to POR measured from the first postoperative ileocolonoscopy. Cox proportional hazard models were used to assess the association of IAUs with time to POR. The mucosa-associated microbiome at first ileocolonoscopy was assessed by sequencing the 16S rRNA gene using biopsies taken from both sides of the anastomosis.

**Results:**

Sixty patients were included, of whom 27 patients had IAUs (45.0%) at first ileocolonoscopy. Median time to first postoperative ileocolonoscopy was 6.5 months (interquartile range [IQR] 5.3-8.1). During a median follow-up duration of 3.0 years (IQR 1.4-5.5) after first postoperative ileocolonoscopy, POR was observed in 53.3%. After adjustment for clinical risk factors, IAUs were independently associated with POR (adjusted hazard ratios 5.4; 95% CI 2.4-12.4; *P* < .001). At the ileal and colonic side of the anastomosis, a significantly higher abundance of *Klebsiella* was associated with IAUs (*q* < 0.05).

**Conclusions:**

IAUs in CD patients with otherwise healthy neo-TI at first postoperative ileocolonoscopy are associated with long-term POR. In addition, a differential mucosa-associated microbiome composition was observed in patients with IAUs, specifically the proteobacteria *Klebsiella*, suggesting that putative taxa are related to these lesions. Further validation studies in larger cohorts, along with mechanistic studies, are still required.

**Key Messages**

**What is already known?**

Isolated anastomotic ulcers (IAUs) are commonly observed in postoperative Crohn’s disease (CD) patients. Their origin remains unclear and may reflect early disease recurrence or a wound-healing response due to post-surgical ischemia. However, their clinical significance and underlying pathophysiology are currently poorly understood.

**What is new here?**

In CD patients with otherwise healthy neo-terminal ileum, the presence of IAUs at the first postoperative ileocolonoscopy is associated with long-term postoperative recurrence. Additionally, a distinct mucosa-associated microbiome profile was identified in patients with IAUs, specifically enrichment of the proteobacteria *Klebsiella*, suggesting potential microbial involvement in the pathogenesis of anastomotic ulcers.

**How can this study help patient care?**

Our findings support the notion that IAUs are an early manifestation of disease recurrence rather than merely a benign healing phenomenon. Although larger studies are needed to validate these findings, patients with IAUs and an otherwise normal neo-terminal ileum may benefit from tight monitoring and proactive disease management to mitigate the risk of recurrence.

## Introduction

Patients with Crohn’s disease (CD) are at considerable risk of undergoing intestinal surgery, as approximately 1 out of 4 patients with CD will undergo an intestinal resection within 10 years of disease diagnosis.^1^ Although intestinal surgery can induce disease remission and improve CD-related symptoms, postoperative recurrence (POR) following ileocolic resection (ICR) is common within 1 year postoperatively.^2^

Early endoscopic assessment (<1 year following ICR) is considered the gold standard for the detection of POR.^3,4^ The original Rutgeerts’ score was established to assess the severity of inflammation in the neo-terminal ileum (TI) and at the ileocolic anastomosis by stratifying the endoscopic severity in 5 groups (i0-i4).^5^ The modified Rutgeerts’ score (mRS) was proposed to differentiate i2 into lesions confined to the anastomosis (i2a) and ≥5 aphthous lesions in the neo-TI (i2b).^6^

Anastomotic ulcers are prevalent in a substantial proportion of patients with CD.^7^ The origin of anastomotic lesions is still unknown and may be related to the wound-healing phenomenon due to post-surgical ischemia or a manifestation of true disease recurrence.^7,8^ Furthermore, the clinical relevance of the lesions is unclear due to conflicting outcomes in the current literature.^9–18^ These conflicting outcomes may be explained by the fact that these studies predominantly compared the long-term outcomes of patients with anastomotic lesions (i2a) to patients with ileal inflammation (i1 or i2b).^10–15^ As patients with i2a lesions may also have concomitant mild ileal inflammation (i1), according to the mRS, the impact of isolated anastomotic ulcers (IAUs) (ie, anastomotic lesions without ileal inflammation) is currently understudied. Additionally, analyses of the mucosal microbiome may aid in understanding the underlying pathophysiology of IAUs development. Therefore, this study aimed to assess the POR risk and the mucosa-associated microbiome composition after ICR in CD patients with and without IAUs among those with otherwise healthy neo-TI at the first postoperative ileocolonoscopy.

## Methods

### Patient Selection, Endoscopic Assessment, and Clinical Data Collection

Patients with CD undergoing ICR with a primary anastomosis at Mount Sinai Hospital (Toronto, Canada) were identified from a prospective study.^19,20^ Exclusion criteria included the absence of ileal disease prior to surgery, isolated small bowel resection with an intact ileocecal valve, (near) subtotal colectomy, or more than 2 prior intestinal resections. Patients were also excluded if the first postoperative ileocolonoscopy was performed before 3 months or after 18 months postoperatively. A less stringent cutoff point of <18 months was chosen as this current study was initiated in 2010, while the international guidelines, recommending to perform an ileoconoscopy within 1 year, were published in 2017 or later.^3,4,21^ The decision to initiate postoperative prophylactic medication and the timing of ileocolonoscopies were left to the discretion of the treating gastroenterologists. However, patients prescribed antibiotic preventive therapy (ie, metronidazole) were excluded.

**Figure 1.**
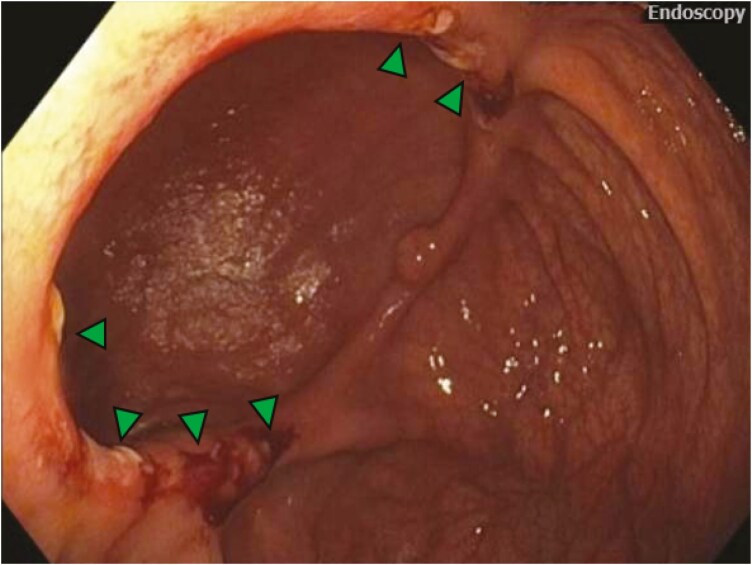
Representative image of isolated anastomotic ulcers during postoperative ileocolonoscopy.

One blinded trained central reader (P.A.O.) reviewed the endoscopic images of the postoperative colonoscopy of each included patient, who underwent ICR at our tertiary referral center, from the ongoing prospective study.^19,20^ Only patients without any macroscopic ulcerations in the neo-TI (SES-CD ≤ 2) at the first postoperative ileocolonoscopy were included in this study. These cases were then assessed for the presence of IAUs at the time of the first postoperative ileocolonoscopy. IAUs were defined as ulcer(s) limited to the anastomotic ring with a minimum size of 5 mm (Figure 1). Ambiguous cases were discussed, and conflicts were resolved with a second blinded reader (M.B.). Eligible cases were subdivided into groups according to the presence or absence of IAUs. Clinical data (demographic, pre-, peri-, and postoperatively) were collected at recruitment and follow-up visits by patient interview and chart review up to January 2025.

### Study Outcome

The primary outcome of the study was time to POR, defined as clinical recurrence (ie, worsening of symptoms) or a change in CD treatment (ie, initiation or dose escalation) accompanied by objective evidence of disease activity within 6 months, need for endoscopic balloon dilation or an intestinal re-resection. Objective evidence of disease activity included C-reactive protein ≥5 mg/L, fecal calprotectin ≥250 µg/g, imaging findings consistent with active inflammation (as assessed by the local radiologist on ultrasound, magnetic resonance imaging, or computed tomography) or endoscopic recurrence (mRS ≥ i2b).

### Sample Collection and 16S rRNA Gene Sequencing

Endoscopists were instructed to take biopsy samples with standard biopsy forceps from the neo-TI between 5 and 10 cm proximal to the anastomosis, colonic mucosa between 5 and 10 cm distal to the ileocolic anastomosis and rectosigmoid area at 20 cm proximal to the anal verge at each postoperative colonoscopy. Samples were snap-frozen in liquid nitrogen and subsequently stored at −80°C until processing. DNA was extracted using the DNeasy 96 PowerSoil Pro QIAcube HT Kit (Qiagen, Germantown, MD) according to the manufacturer’s instructions. The V3-V4 hypervariable regions of the 16S rRNA gene were polymerase chain reaction amplified using Illumina MiSeq (v3 Kit) to produce 2 × 300 bp sequencing products.^22^ Demultiplexed FASTQ files were processed using the Quantitative Insights Into Microbial Ecology 2 (QIIME2) pipeline, and a matrix of amplicon sequence variants was generated.^23^ Sequences were taxonomically classified using the Silva (release 138) reference database.^24^ For this study, we assessed the mucosa-associated microbiome using the samples taken proximal and distal from the ileocolic anastomosis.

### Statistical Analyses

All data preparation and downstream analysis were performed in R software version 4.2.2. Descriptive statistical analysis (frequency, percentage, mean, standard deviation [SD], median, and interquartile range [IQR]) was used to describe the analytical sample. Categorical variables were quoted as number and percentage. Continuous variables were presented as median and IQR or mean and SD, depending on their distribution. Baseline and disease-related characteristics were compared between patients with and without IAUs using the chi-square test for categorical data, and the Mann–Whitney test or *t*-test for continuous data. Missing data were handled using multiple imputation by chained equations, with 5 imputed datasets generated using the package.^26^ Kaplan–Meier curves, including log-rank test for significance, were used to describe and compare survival probabilities between the 2 groups (those with or without IAUs). The association of IAUs and the time-to-event outcome was investigated using cause-specific Cox proportional hazard models after adjusting for a priori-defined clinical confounding factors (active smoking, prior intestinal resection, and treatment at first postoperative ileocolonoscopy). The time to event was defined as the time between the first postoperative ileocolonoscopy and the study outcome (described above). Patients who did not experience the respective event were censored at last follow-up. Results from the proportional hazards models are presented as adjusted hazard ratios (aHR) and corresponding 95% confidence intervals (CI). As a sensitivity analysis, an alternative variable selection approach was applied, whereby variables with a *P*-value < .10 in univariate analysis were included in the multivariable model.

### Microbiome Analysis

Microbiome analysis was performed on samples with more than 3000 reads and in patients not treated with antibiotics at the time of index postoperative colonoscopy. Microbiome analysis in this cohort was earlier described by Hernandez-Rocha et al.^20^ Micriobiome analyses wereperformed separately at neo-TI and ascending colon. Alpha diversity was determined on aggregated microbial amplicon sequence variants with identical genus taxonomic classification. After assessing the rarefaction curve, rarefaction at a depth of 8000 reads per sample was applied using package.^27^ Alpha diversity was calculated using the Chao1 index and Shannon’s diversity index, and compared between patients with and without IAUs using the Wilcoxon’s rank sum test. For beta diversity analysis, after the removal of singletons, principal coordinate analysis was performed based on the Bray–Curtis dissimilarity index at the genus level. Permutation analysis of variance was applied using the adonis function of the package on distance matrices with 1000 permutations.^28^

We performed multivariable association analysis between microbial genus-level relative abundances and clinical covariates using the MaAsLin2 (Multivariate Association with Linear Models) R package (version 1.12.0).^29^ Taxonomic profiles were normalized by total sum scaling without further transformation. Associations were assessed using a compound Poisson linear model, adjusting for the fixed effects of anastomotic ulcer status, age at index surgery, and sex. We reported adjusted *q* values <0.20 using Benjamini–Hochberg correction for multiple testing, and *q*-values <0.05 were considered significant.

### Ethics

Informed consent was obtained from every participant prior to the inclusion of the study. The study was approved by the institutional research ethics board approval at Mount Sinai Hospital Toronto (10-0243-E).

## Results

### Patient Demographics

In total, 162 patients, who underwent an ICR between June 2011 and November 2020, were included in the prospective study, of which 60 (37.0%) met eligibility criteria for this analysis (Table 1). The median follow-up time of the study cohort was 3.0 years (IQR 1.4-5.5). Twenty-eight patients (46.7%) were male. The median age and disease duration at index surgery were 32.5 years (IQR 26.0-41.5) and 9.0 years (IQR 2.0-16.5). Ten patients (16.7%) were active smokers at the time of surgery, 19 patients (31.7%) underwent a prior intestinal resection, and 29 patients (48.3%) had prior exposure to at least one biologic agent preoperatively. The most common disease localization and behavior at the time of surgery, according to the Montreal classification, were ileocolic (61.7%, *n* = 37) and penetrating disease (60.0%, *n* = 36). The time to first postoperative ileocolonoscopy was 6.5 months (IQR 5.7-8.6) from index surgery. At the first postoperative ileocolonoscopy, 45.0% (*n* = 27) of the patients had IAUs. 49.3% (*n* = 29) of the patients were on treatment at the time of the first postoperative ileocolonoscopy, including immunomodulator monotherapy (13.8%), biologic monotherapy (69.0%), or combination therapy (17.2%) (ie, immunomodulator with a biological). No significant differences were observed in the baseline characteristics between patients with and without IAUs (Table 1).

**Table 1.** Baseline characteristics of the study cohort (*n* = 60).

Variables	Total study population (*n* = 60)	Patients with no isolated anastomotic ulcers (*n* = 33)	Patients with isolated anastomotic ulcers (*n* = 27)	*P*-value
Age at surgery (years), median (IQR)	32.5 (26.0-41.5)	33.0 (27.0-45.0)	32.0 (23.5-38.0)	.29
Sex (male), *n* (%)	28 (46.7)	15 (45.5)	13 (48.1)	>.99
Disease duration at surgery (years), median (IQR)	9.0 (2.0-16.5)	9.0 (2.0-19.0)	10.0 (4.5-14.5)	.88
Active smoker, *n* (%)	10 (16.7)	4 (12.1)	6 (22.2)	.49
Age at diagnosis (Montreal classification), *n* (%)				.67
A1 (<17 years)	14 (23.3)	7 (21.2)	7 (25.9)	
A2 (17-40 years)	41 (68.4)	24 (72.7)	17 (63.0)	
A3 (>40 years)	5 (8.3)	2 (6.1)	3 (11.1)	
Disease localization at surgery (Montreal classification), *n* (%)				.13
L1 (ileal)	23 (38.3)	16 (48.5)	7 (25.9)	
L3 (ileocolic)	37 (61.7)	17 (51.5)	20 (74.1)	
+L4 (upper gastrointestinal disease)	5 (8.3)	2 (6.1)	3 (11.1)	.81
Disease behavior at surgery (Montreal classification), *n* (%)				.22
B2 (stenotic)	24 (40.0)	16 (48.5)	8 (29.6)	
B3 (penetrating)	36 (60.0)	17 (51.5)	19 (70.4)	
+p (perianal)	14 (23.3)	6 (18.2)	8 (29.6)	.46
Prior intestinal resection, *n* (%)	19 (31.7)	12 (36.4)	7 (25.9)	.56
Side-to-side anastomosis, *n* (%)	51 (91.1)	28 (90.3)	23 (92.0)	.96
Length of resection specimen (in cm), mean (SD)	25.3 (14.1)	24.3 (14.5)	26.6 (13.8)	.52
Free surgical margins, *n* (%)	50 (89.3)	27 (87.1)	23 (85.2)	.11
Preoperative biologic exposure, *n* (%)	29 (48.3)	19 (59.4)	10 (37.0)	.15
Time to first postoperative ileocolonoscopy (months), median (IQR)	6.5 (5.7-8.6)	6.7 (5.4-8.2)	6.2 (5.2-8.0)	.57
Treatment at first postoperative ileocolonoscopy, *n* (%)	29 (49.3)	18 (54.7)	11 (40.7)	.29
Immunomodulator monotherapy	4 (6.7)	2 (6.1)	2 (7.4)	.84
Biologic monotherapy	20 (33.3)	13 (39.4)	7 (25.9)	.27
Combination therapy (immunomodulator and biological)	5 (8.3)	3 (9.1)	2 (7.4)	.81

Abbreviations: IQR, interquartile range. In case of missing data, valid rates are presented.

### Long-Term POR

**Figure 2.**
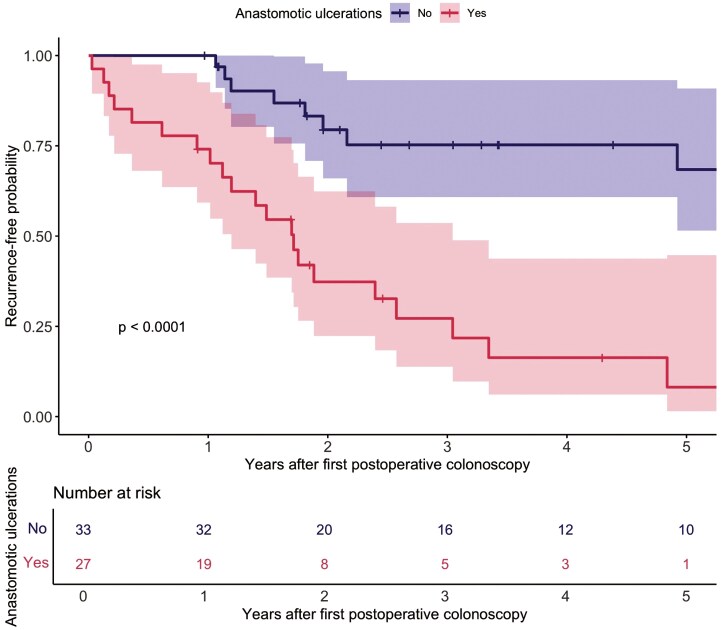
Kaplan–Meier analysis of postoperative recurrence in patients with Crohn’s disease after ileocolic resection according to the presence of isolated anastomotic ulcers at first postoperative ileocolonoscopy.

Long-term POR was observed in 32 patients (53.3%) after a median interval of 1.7 years (IQR 1.0-3.3) from the first postoperative ileocolonoscopy to the first episode of long-term POR. A significantly shorter time to POR was observed in patients with IAUs at first postoperative ileocolonoscopy as compared to those without IAUs (log-rank test *P* < .01)(Figure 2). During the study period, clinical recurrence was observed in 25/60 patients (41.7%, 20/27 with IAUs [74.1%] vs. 15/33 without IAUs [45.5%]), change in IBD-related therapy in 28/60 patients (46.7%, 17/27 with IAUs [63.0%] vs. 11/33 without IAUs [33.3%]), endoscopic balloon dilatation in 2/60 patients (3.3%, 1/27 with IAUs [3.7%] vs. 1/33 without IAUs [3.0%]) and surgical recurrence in 4/60 patients (6.7%, 3/27 with IAUs [11.1%] vs. 1/33 without IAUs [3.0%]).

After adjustment for clinical risk factors (active smoking, prior intestinal resection, and treatment at first postoperative ileocolonoscopy), IAUs were independently associated with POR (aHR 5.46; 95%CI 2.40-12.41; *P* < .001)(Table 2). Results were driven by clinical recurrence and change in IBD-related therapy (Supplementary Figure S1). As a sensitivity analysis, we performed an alternative variable selection strategy, in which variables with a *P*-value <.10 in the univariate analysis were included in the multivariate Cox model. Results were consistent with the primary analysis (Supplementary Table S1), showing an independent association for IAUs with POR (aHR 5.43; 95%CI 2.30-12.84; *P* < .001). Furthermore, L4 disease (according to the Montreal classification) (aHR 3.82; 95%CI 1.36-10.75; *P* = .017). and immunomodulator monotherapy at first postoperative ileocolonoscopy (aHR 10.39; 95%CI 2.60-42.31; *P* = .003) were associated with POR.

**Table 2.** Multivariable Cox proportional hazard model based on a priori-defined confounders.

Histopathological variables	HR	95% CI	*P*-value
*Isolated anastomotic ulcers*	*5.46*	*2.40*-*12.41*	*<.001*
Active smoking	0.94	0.41-2.16	.884
Prior intestinal resection	1.17	0.49-2.81	.723
Treatment at first postoperative ileocolonoscopy	1.21	0.57-2.54	.625

### Microbiome Composition in Patients With and Without IAUs

**Figure 3.**
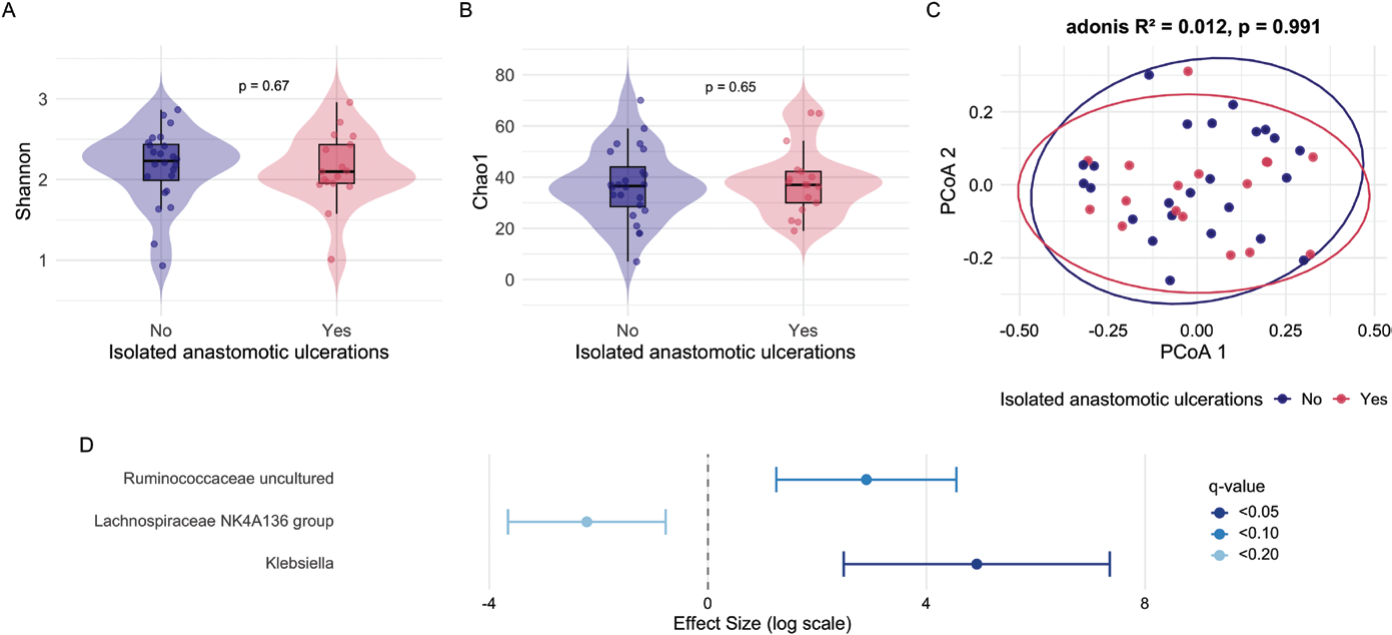
Analysis of the mucosa-associated microbiota from the neo-terminal ileum (*n* = 41) comparing patients with and without isolated anastomotic ulcers. The figure includes alpha diversity metrics (A and B), beta diversity based on Bray–Curtis dissimilarity index (C), and differential relative abundance at the genus level (D).

**Figure 4.**
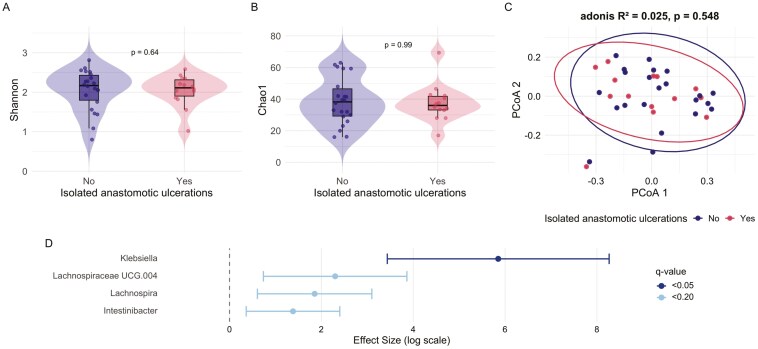
Analysis of the mucosa-associated microbiota from the ascending colon (*n* = 36) comparing patients with and without isolated anastomotic ulcers. The figure includes alpha diversity metrics (A and B), beta diversity based on Bray–Curtis dissimilarity index (C), and differential relative abundance at the genus level (D).

Samples from both proximal and distal of the ileocolic anastomosis were obtained from 41 patients (68.8%, 24 without IAUs, 17 with IAUs) at the time of first postoperative ileocolonoscopy. For diversity analysis, 5 of the 41 colon samples were discarded after rarefaction, leaving 36 samples (22 no IUAs, 14 with IUAs). No differences were observed in alpha or beta diversity between patients with and without IAUs at the neo-TI and colon (Figures 3 and 4). Analysis of the samples from the neo-TI showed a significantly higher abundance of genus *Klebsiella* in patients with IAUs (coefficient = 4.92, standard error [SE] = 1.24, q = 0.04) (Figure 2). In addition, a trend of a higher abundance of genus *Ruminococcaceae uncultured* (coefficient = 2.90, SE = 0.84, *q* = 0.09) and a lower abundance of genus *Lachnospiraceae NK4A136 group* (coefficient=−2.22, SE = 0.74, *q* = 0.17) was observed in patients with IAUs. Analysis of the samples obtained from the ascending colon showed a significantly higher abundance of *Klebsiella* in subjects with IAUs (coefficient = 5.85, SE = 1.23, *q* = 0.006) (Figure 3). Furthermore, a higher abundance was observed for *Lachnospiraceae UCG004* (coefficient = 2.30, SE = 0.80, *q* = 0.12)*, Lachnospira* (coefficient = 1.85, SE = 0.64, *q* = 0.12) and *Intestinibacter* (coefficient = 1.38, SE = 0.52, *q* = 0.19) in patients with IAUs.

After assessing the microbial diversity and the abundance between patients experiencing long-term POR versus no long-term POR, we found no differences in diversity between these 2 groups. A significant higher abundance of *Klebsiella* in both the samples from the colon (coefficient = 7.16, SE = 1.61, *q* < 0.01) (Supplementary Figure S2) and neo-TI (coefficient = 24.28, SE = 5.61, *q* = 0.02) (Supplementary Figure S3) was observed in those experiencing long-term POR as compared to those who did not.

## Discussion

This study evaluated the long-term clinical impact of IAUs without inflammation in the neo-TI. Our outcomes showed a significantly shorter time to POR during the study period. Furthermore, IAUs were found to be independently associated with POR by multivariable analysis. In addition, a differentially abundant mucosa-associated microbiome composition, with a distinct higher abundance of *Klebsiella*, was observed in patients with IAUs as compared to those without IAUs, which may suggest a role for this taxa in isolated anastomotic lesions. This distinct higher abundance was also observed in those who developed POR.

Conflicting results in the current literature have been reported with regard to the clinical relevance of the IAUs.^9–18^ These conflicting outcomes may be explained by the fact that these studies predominantly compared the long-term outcomes of patients with anastomotic lesions (i2a) to patients with ileal inflammation (i1 or i2b).^10–15^ As patients with i2a lesions may also have concomitant mild ileal inflammation (i1), which confers an increased risk of long-term clinical recurrence and progression to severe endoscopic recurrence as compared to i0, it is unknown whether the lesions on the anastomosis, neo-TI, or both contribute to the disease recurrence.^9^ As the mRS does not allow for separately assessing the lesions at the ileocolic anastomosis and the neo-TI, the prognosis of IAUs is currently understudied. An endoscopic score assessing the lesions at separate anastomotic locations seems more appropriate to study the prognosis and relevance of lesions in these specific anatomic areas.^16,30^

The pathogenesis of IAUs is still unclear and may be related to disease recurrence or a wound-healing phenomenon due to post-surgical ischemia. A recent study from Italy reported a presumed ischemic ulcer prevalence of 19%.^31^ A Dutch study reported a high rate of ulcerations (77%) on the inverted stapled line (ie, at the ileocolic anastomosis) in patients with CD, whereas ulcerations on the everted stapled line (ie, at the colonic blind loop) were nearly absent (<2%).^7^ Likewise, ulcerations on the inverted stapled line were prevalent in the majority of patients with colorectal cancer (68%) following right-sided hemicolectomy. No ulcerations on the everted stapled line were observed in patients with colorectal cancer. Based on these outcomes, the authors suggest a difference in mechanism with mucosa-to-mucosa adaptation, on the everted stapled line, resulting in primary wound healing whereas a serosa-to-serosa adaptation, on the inverted stapled line (ileocolic anastomosis), will lead to secondary healing with ulcerations due to the need for re-epithelialization and due to ischemia caused by the surgical staples.^7,32^

In contrast to these findings, a histopathological pilot study reported Crohn-like features in the vast majority (93%) of the anastomotic mucosal specimens (29 specimens in 8 patients, including targeted endoscopic biopsies from the anastomosis and/or an ICR specimen), accounting for all of the included patients.^8^ Only 2 specimens, accounting for 2 patients, also had ischemic-like features in these specimens. These findings, supporting that anastomotic ulcers are a manifestation of disease recurrence, are in line with the observations in this current study. Furthermore, 2 large Asian studies reported an independent association of more severe anastomotic lesions with endoscopic recurrence or subsequent interventions, including endoscopic balloon dilatation or intestinal re-resection.^14,17^ In addition, Hammoudi et al. observed a significantly shorter time to clinical recurrence in patients with at least semi-circumferential anastomotic lesions as compared to patients with no to mild anastomotic lesions.^16^ These outcomes suggest that at least a subset of anastomotic lesions are a manifestation of disease recurrence, instead of merely a wound healing phenomenon, as these lesions contribute to the long-term POR risk.^14,16,17^ However, further research is warranted to study the pathophysiology of these lesions to differentiate which type of ulcers are a post-surgical ischemic phenomenon or true disease recurrence.

One related hypothesis is that POR in CD is dependent on the continuity of the fecal stream, which contributes to the role of the microbiome in the development of disease recurrence after surgery.^33^ Several individual bacterial taxa, found in the resection specimen, neo-TI, or feces, have been identified to be associated with (severe) ePOR.^34^ Our study is the first to assess and compare the mucosa-associated microbiome composition in patients with or without IAUs in a healthy neo-TI. We observed a distinct composition between these 2 groups (IAUs vs. no IAUs but also POR vs. no POR), specifically a significantly higher abundance of *Klebsiella* was found on both sides of the ileocolic anastomosis. An increase in the proinflammatory proteobacteria *Klebsiella* has been associated with disease relapse and severity of symptoms in patients with inflammatory bowel disease.^35,36^ In addition, *Klebsiella* species are recognized as pathobionts and may contribute to the exacerbation of inflammatory bowel disease in genetically susceptible individuals.^25^ Furthermore, a higher abundance of *Klebsiella* was found to be associated with future endoscopic recurrence (≥i2) in patients in endoscopic remission (i0-i1) at first postoperative ileocolonoscopy in our recently published multicenter cohort study.^20^ The outcomes of Hernandez-Rocha et al., which investigated a larger number of patients who had no to mild lesions in the neo-TI at the first postoperative colonoscopy, suggest a role for the mucosa-associated microbiome, especially *Klebsiella*, for triggering disease recurrence in CD in a subsequent postoperative colonoscopy.^20^ It is important to note that our study by Hernandez-Rocha et al. and the current study have been performed within the same cohort.^20^ These results suggest that the microbiome at the first postoperative ileocolonoscopy may be used as a biomarker for future disease recurrence and to potentially target the microbiome composition in order to prevent disease recurrence.

This study is the first to assess the impact of IAUs on long-term POR and to study the mucosa-associated microbial composition in CD patients with otherwise healthy neo-TI at first postoperative ileocolonoscopy following ICR. The strengths of our study include the prospective study design, the long-term follow-up, the use of central reading to assess the presence or absence of the IAUs, and the use of objective parameters of POR. Nevertheless, we were limited by our relatively small sample size. Therefore, we applied strict statistical approaches such as using an a priori model in our primary analysis with the most consistent confounding factors for POR to prevent overfitting.^29^ The small sample size, but also the low rates of active smoking and medication use at the time of the first postoperative colonoscopy in our cohort, may have contributed to the lack of a demonstrated association of these variables. However, the rates of these variables are in line with our larger cohort.^19^ Furthermore, the predesigned protocol for biopsy collection did not include biopsies from the ileocolic anastomosis itself due to concerns of bleeding; however, the mucosal-associated microbiome from both the neo-TI and colonic biopsies, 10 cm proximal to and distal to the anastomosis, may be representative of the microbiome environment at the anastomosis. In addition, endoscopic characteristics of the ulcers, such as ulcer size, depth, or circumferential involvement, or histological activity in the obtained biopsies, were not reported in a standardized manner, which did not allow us to study a potential correlation between the endoscopic severity of IAUs or histological activity and our outcomes.

## Conclusion

IAUs in CD patients with a healthy neo-TI at first postoperative ileocolonoscopy are associated with long-term POR. In addition, a differential mucosa-associated microbiome composition was observed in patients with IAUs, specifically the proinflammatory pathobiont *Klebsiella*, suggesting that this taxa may be related to these lesions. Further validation studies in larger cohorts, along with mechanistic studies, are still required.

## Supplementary data

Supplementary data is available at *Inflammatory Bowel Diseases* online.

izaf147_Supplementary_Figures_1-3_Tables_1

## Data Availability

The data underlying this article will be shared on reasonable request to the corresponding author.
