# Supplementary material for: Isolated Anastomotic Ulcers Are Associated with a Higher Long-Term Risk for Postoperative Recurrence and a Differential Mucosa-Associated Microbiome Composition in Patients with Crohn’s Disease Following Ileocolic Resection
Source: Inflamm Bowel Dis. 2025 Jul 16;31(12):3247–55. doi: 10.1093/ibd/izaf147 (PMC12688075; doi:10.1093/ibd/izaf147)
Supplement: izaf147_Supplementary_Figures_1-3_Tables_1 [file izaf147_supplementary_figures_1-3_tables_1.docx]

**Supplementary Files**

**Supplementary Table 1.** Sensitivity analysis

|  | **Univariate analysis** | | | | **Multivariate analysis** | | | |
| --- | --- | --- | --- | --- | --- | --- | --- | --- |
| Variable | HR | Lower 95%CI | Upper  95%CI | p-value | HR | Lower  95%CI | Upper  95%CI | p-value |
| **Anastomotic ulcerations** | **5.00** | **2.28** | **10.93** | **<0.001** | **5.43** | **2.30** | **12.84** | **<0.001** |
| Age at index surgery | 1.00 | 0.97 | 1.03 | 0.98 |  |  |  |  |
| Sex (male) | 1.02 | 0.51 | 2.02 | 0.97 |  |  |  |  |
| Disease duration at index surgery | 1.01 | 0.98 | 1.04 | 0.61 |  |  |  |  |
| Active smoking | 1.13 | 0.51 | 2.53 | 0.76 |  |  |  |  |
| Montreal Classification - Age A2 (versus A1) | 0.78 | 0.36 | 1.69 | 0.52 |  |  |  |  |
| Montreal Classification - Age A3 (versus A1) | 0.49 | 0.06 | 3.94 | 0.50 |  |  |  |  |
| Montreal Classification - Location Ileocolic (versus Ileal) | 1.08 | 0.51 | 2.30 | 0.84 |  |  |  |  |
| **Montreal Classification - Location L4** | **2.71** | **1.04** | **7.10** | **0.04** | **3.82** | **1.36** | **10.75** | **0.017** |
| Montreal Classification - Behavior fistulizing (versus stricturing) | 0.99 | 0.47 | 2.06 | 0.97 |  |  |  |  |
| Montreal Classification - Perianal | 1.83 | 0.88 | 3.80 | 0.11 |  |  |  |  |
| Penetrating Disease (B3 or perianal) | 0.99 | 0.47 | 2.06 | 0.97 |  |  |  |  |
| Prior intestinal resection | 0.85 | 0.38 | 1.90 | 0.68 |  |  |  |  |
| Preoperative biologic exposure | 0.65 | 0.33 | 1.31 | 0.23 |  |  |  |  |
| IBD treatment at first postoperative colonoscopy | 0.82 | 0.41 | 1.63 | 0.57 |  |  |  |  |
| **IMM monotherapy at first postoperative colonoscopy** | **3.82** | **1.12** | **13.03** | **0.03** | **10.39** | **2.60** | **42.31** | **0.003** |
| Anti-TNF monotherapy at first postoperative | 1.08 | 0.51 | 2.27 | 0.84 |  |  |  |  |
| Other biologic at first postoperative colonoscopy | 0.43 | 0.06 | 3.17 | 0.41 |  |  |  |  |
| Combination therapy at first postoperative colonoscopy | 0.00 | 0.00 | Inf | 1.00 |  |  |  |  |
| Any biologic therapy at first postoperative colonoscopy | 0.91 | 0.44 | 1.88 | 0.80 |  |  |  |  |
| Time from index surgery to first postoperative | 1.00 | 1.00 | 1.00 | 0.20 |  |  |  |  |
| Length of resection | 1.01 | 0.99 | 1.04 | 0.28 |  |  |  |  |
| Side-to-side anastomosis | 1.53 | 0.38 | 6.07 | 0.55 |  |  |  |  |
| Free margins | 0.45 | 0.15 | 1.31 | 0.15 |  |  |  |  |
| **BMI** | **0.94** | **0.87** | **1.01** | **0.08** | 0.96 | 0.90 | 1.03 | 0.260 |

**Supplementary Figure 1.** Hazard ratios with 95% confidence intervals for individual outcomes of the primary composite outcome

**
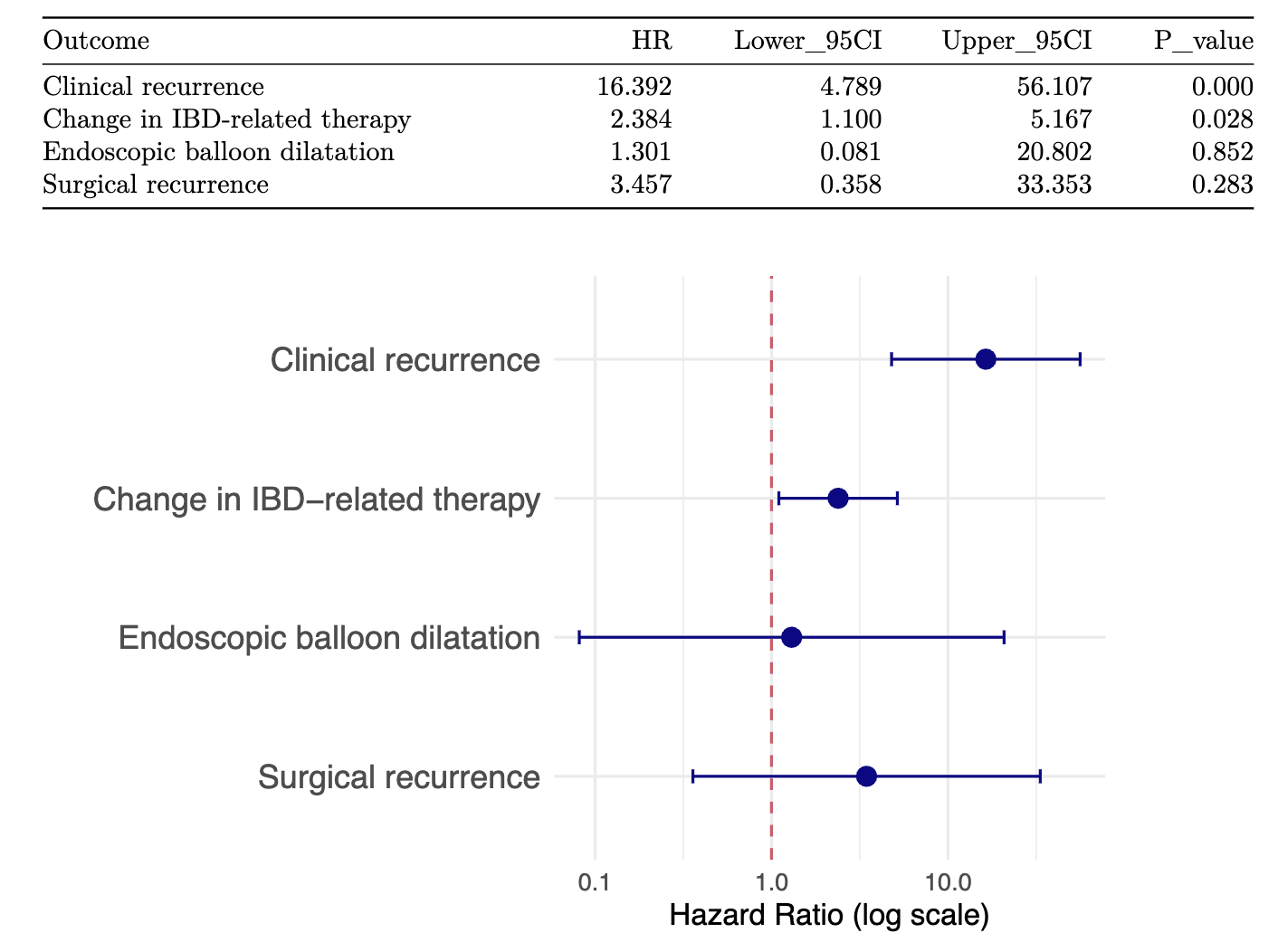
**


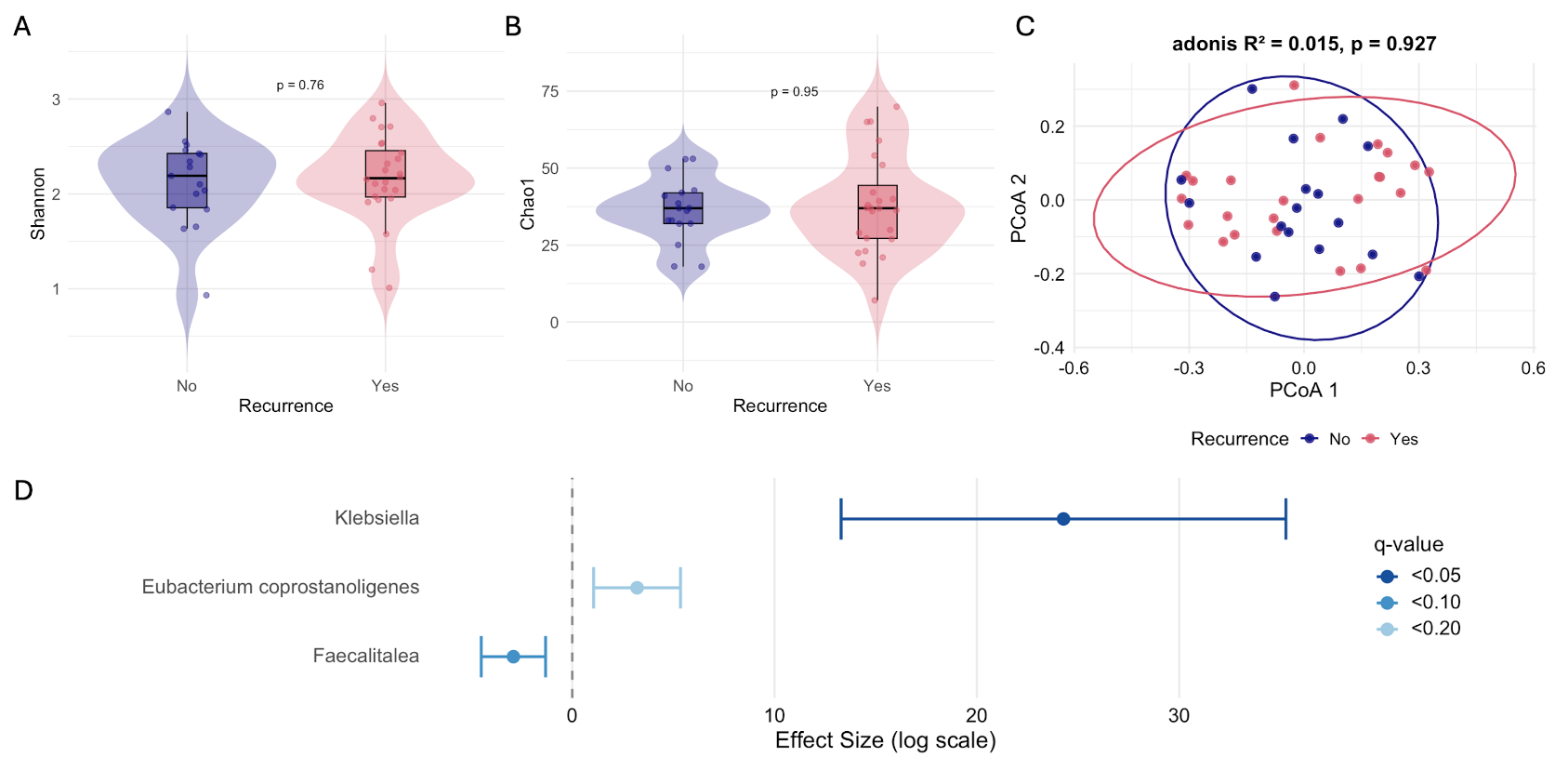
**Supplementary Figure 2.** Analysis of the mucosa-associated microbiota from the neoterminal ileum (n=41) comparing patients experiencing postoperative recurrence versus those not experiencing postoperative recurrence. The figure includes alpha diversity metrics (Panels A and B), beta diversity based on Bray-Curtis dissimilarity index (Panel C), and differential relative abundance at the genus level (Panel D).


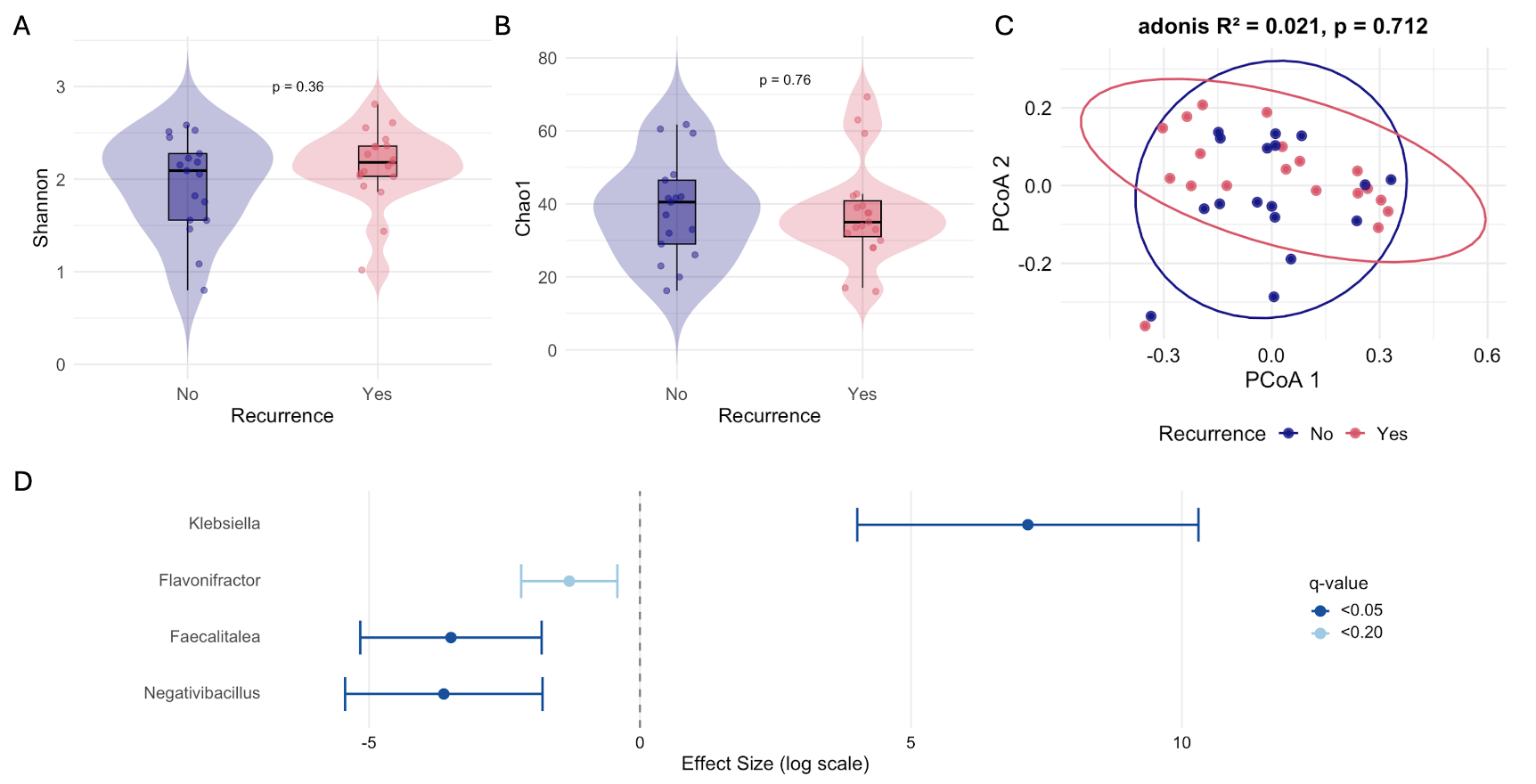
**Supplementary Figure 3.** Analysis of the mucosa-associated microbiota from the ascending colon (n=36) comparing patients experiencing postoperative recurrence versus those not experiencing postoperative recurrence. The figure includes alpha diversity metrics (Panels A and B), beta diversity based on Bray-Curtis dissimilarity index (Panel C), and differential relative abundance at the genus level (Panel D).
